# Housing Environmental Enrichment, Lifestyles, and Public Health Indicators of Neurogenesis in Humans: A Pilot Study

**DOI:** 10.3390/ijerph21121553

**Published:** 2024-11-25

**Authors:** Mohamed Hesham Khalil, Koen Steemers

**Affiliations:** Department of Architecture, University of Cambridge, Cambridge CB2 1PX, UK; kas11@cam.ac.uk

**Keywords:** neuroplasticity, environmental enrichment, housing design, home, built environment, mental health, cognitive reserve, brain health, physical activity, adult hippocampal neurogenesis, neuroarchitecture

## Abstract

Background: In response to the rising mental health concerns and cognitive decline associated with the human brain’s neurogenesis, which continues until the tenth decade of life but declines with age and is suppressed by poor environments, this pilot study investigates how physical environments may influence public health proxy measures of neurogenesis in humans. This pilot study focuses on the residential environment where people spend most of their time and age in place, exploring the dependency of depression, anxiety, and cognitive impairment variations on spatial and lifestyle variables. Methods: A total of 142 healthy adults in England completed a survey consisting of PHQ-8, GAD-7, and CFI questionnaires and other questions developed to capture the variance in spatial and lifestyle factors such as time spent at home, house type layout complexity, spaciousness, physical activity, routine and spatial novelty, and perceived loneliness. Results: Extensive time spent at home has adverse effects on all measures, while multi-storey houses perform better than single-story houses with positive correlations with physical activity and spatial novelty. Separate regression models on the variance in depression, as the most salient dependent variable and reliably associated with neurogenesis, reveal that getting out of the house explains 20.5% of the variance in depression symptoms. At the scale of the house, multi-storey houses explain 16.5% of the variance. Both percentages are closer to the effect of loneliness, which we found to explain 26.6% of the variance in depression. Conclusions: The built environment appears to be significantly associated with changes in cognitive function and mental health symptoms associated with neurogenesis. This pilot study shows the equally important effect of physical and social enrichment, offering critically needed insights for neuroarchitecture and brain health research that is interested in public health.

## 1. Introduction

With the increasing research gap on environmental enrichment and neuroplasticity in humans, alongside rising mental health disorders, environmental neuroscience seeks to explore the connection between the physical environment and mental health [1], and explore how the physical environment can promote neurosustainability by sustaining neuroplasticity through the environment for improved cognition and health [2]. Given that people spend most of their time at home [3], this pilot study investigates the relationships between housing environmental enrichment, lifestyle variables, and proxy measures of adult hippocampal neurogenesis (AHN) in the human brain. AHN is the continuous generation of new neurons, leading to improvements in mood and cognition [4]. What makes AHN vital is that it persists into the tenth decade of life [5,6], but it declines with ageing [7], and is influenced by the environment [8]. This pilot study uses depression, anxiety, and cognitive impairment as the three are representative of AHN, as will be explained shortly, and they are also of interest in public health research [9,10,11,12,13]. Therefore, the insights to be derived from this pilot study can benefit both healthy individuals and those with cognitive or mental health conditions to whom the residential environment can be a critical driver for their lifelong AHN.

By drawing on the new direction of environmental neuroscience towards exploring the pathway between physical environment ingredients and mental health, this pilot study is contextually designed after the evidence on time spent at home in Britain and the recent evidence on the general adverse effects of spending time at home on mental health. On the one hand, this research aligns with the work of [3], who have raised the multidimensional importance of the home environment and how it can affect well-being and health in many ways. Their work and suggestions were developed after considering the common sedentary behaviours associated with the home environment. They stated that in Britain, for instance, people spend as much as 90% of their time inside buildings and 70% at home. The authors further discussed how designing healthy homes through the house’s architecture, light, air, and more variables can align with sustainability practices and enhance occupant well-being. On the other hand, this pilot study follows up on recent evidence suggesting that getting out of the house is beneficial for people with serious mental illness [14], showing that spending extensive amounts of time at home contributes to poor cognitive function. In that regard, this paper adopts a novel perspective on the markers of a healthy home, focusing on AHN and exploring the relationship between the residential environment and AHN through cognitive and mental health indicators.

This paper aims to offer novel insights into the complex interrelationship between the residential environment and neurogenesis. The contribution of this paper expands beyond its novel perspective to offer insights for several research topics. It contributes to providing explanations for why homebodies were found to have poorer cognitive function [14], and the extent to which the physical home environment during the global COVID-19 pandemic lockdowns and demands to stay at home may have had adverse effects on mental health [15,16,17,18,19,20]. Furthermore, this pilot study aims to bridge the gap in research on environmental enrichment and neuroplasticity between humans and rodents [8,21,22,23,24].

This study uses a sample of participants from England to align with the reporting on the percentages of time spent at home and follow up on the recommendations for designing healthy homes [3]. Still, the findings from this pilot study can be helpful towards more geographic contexts that share similar lifestyle patterns, may be valuable for other building types beyond housing, and aspire to be of interest to promoting brain health and cognitive reserve, public health, and mental well-being.

## 2. Literature Review

### 2.1. Adult Hippocampal Neurogenesis (AHN)

The dentate gyrus (DG), a region within the hippocampus in the brain, continuously undergoes a process called adult hippocampal neurogenesis (AHN) [25]. This neurogenic process involves producing, developing, and incorporating newly generated neurons into the pre-existing neural network. Recent studies have shown that AHN continues throughout life, not only into the eighth but even the tenth decade of a human’s life [5,6,26]. Newborn neurons at various stages of maturation may have unique roles in learning and memory processes [27]. Impairments in this process have been linked to various cognitive and psychological disorders, such as deficits in memory encoding, mood-related conditions, and dementia [28,29].

### 2.2. Depression, Anxiety, and Cognitive Impairment as Public Health Indicators of AHN

Research testing AHN in humans is often invasive and bound by methodological and ethical limitations, unlike research on non-human subjects, which limits research on human subjects. Hence, this study considers depression, anxiety, and cognitive impairment as three proxy measures for AHN in humans, which can help explore any significant relationship evident across the three proxy measures before attempting to use biomarkers or neuroscience methods requiring blood samples or MRI brain scans.

#### 2.2.1. Depression

Depression is a prevalent mental health disorder characterised by persistent feelings of sadness, hopelessness, and loss of interest [30]. However, the symptoms of depression may rise among healthy individuals in response to environmental situations such as during the COVID-19 lockdown [31,32,33]. Evidence suggests that impaired AHN may contribute to the pathophysiology of depression and that increased AHN may modulate dentate gyrus function to provide some behavioural effects of antidepressants [34]. This is known as the neurogenesis hypothesis of depression. For instance, studies have revealed reduced hippocampal volume and decreased neural progenitor cells in the dentate gyrus of individuals with major depressive disorder (MDD) compared to healthy controls [35,36]. Additionally, antidepressant treatment has increased hippocampal volume and promoted AHN in both animal models and humans [37,38]. Functional neuroimaging studies have also provided insights into the role of AHN in depression. A study used functional magnetic resonance imaging (fMRI) to investigate hippocampal activation in patients with a history of major depression. The authors found that patients with a history of depression exhibited reduced hippocampal activation during a memory task compared to healthy controls. The findings suggest that altered hippocampal function may be a trait marker of vulnerability to depression [39]. More recently, Boldrini et al. [40] found that individuals with MDD who died by suicide had fewer granule neurons in the dentate gyrus of the hippocampus compared to controls. In contrast, individuals who had MDD but did not die by suicide had more granule neurons, suggesting a potential role of neurogenesis in resilience to suicide. A study done on rodents by Eliwa et al. [41] found that increasing adult neurogenesis in the hippocampus using a genetic approach reduced anhedonia (loss of pleasure) in a mouse model of chronic stress and depression. The findings suggest that promoting neurogenesis may have therapeutic potential for depression. A most recent systematic review and meta-analysis by Santos et al. [42] investigated global hippocampal atrophy in MDD. The authors found that patients with MDD had significantly smaller hippocampal volumes compared to healthy controls, and this effect was more pronounced in patients with recurrent episodes and a longer duration of illness. Afterwards, it was reported that volumetric changes in the neurofunctional subfield of the hippocampus are prospective markers allowing the prediction of MDD occurrence [43]. The brain-derived neurotrophic factor (BDNF), measured using blood samples, has been studied concerning depression and hippocampal volume. BDNF may contribute to the reduced hippocampal volume observed in some patients with major depression [44]. From the presented evidence, we argue that depression may act as a reliable indicator of reduced AHN for the purpose of this pilot study.

#### 2.2.2. Anxiety

Anxiety disorders, such as generalised anxiety disorder (GAD), are characterised by excessive worry, fear, and avoidance behaviours [30]. Similar to depression, the COVID-19 lockdown in the UK has increased anxiety among the general population [33]. Research suggests that altered AHN may underlie the overgeneralisation thinking patterns often seen in anxiety disorders [45]. Several studies have reported a correlation between decreased AHN and increased anxiety-like behaviour in animals [46,47]. A study by Hill et al. [48] provides valuable insights into the relationship between AHN and both anxiety and depression-related behaviours in mice. The findings suggest that promoting AHN could be a potential strategy for reducing or preventing the symptoms of anxiety and depression, particularly in the context of chronic stress. Impairment of AHN may underlie the pathophysiology of affective disorders, including anxiety, and that neurogenesis-inspired therapies may be a promising approach to reducing symptoms of these disorders in humans [49].

#### 2.2.3. Cognitive Impairment

Several studies have demonstrated that AHN is associated with cognitive performance in healthy adults. Higher levels of AHN correlated with better performance on a pattern separation task [50]. According to Gomes-Leal et al. [49], deficits in AHN might impair normal dentate gyrus function, including pattern separation and cognitive flexibility, which could play a role in the aetiology of affective disorders such as anxiety. This cognitive process relies on the dentate gyrus, as introduced earlier in this paper. To further elaborate, pattern separation is how similar experiences or memories are encoded as distinct representations, allowing for accurate retrieval [51]. Similarly, Clelland et al. [52] showed that inhibiting AHN in mice led to impairments in spatial memory and pattern separation. In addition to its role in healthy cognitive function, AHN has also been implicated in cognitive impairment and neurodegenerative diseases. For instance, post-mortem studies have revealed that individuals with Alzheimer’s disease (AD) exhibit reduced AHN compared to age-matched controls [26]. The relationship between AHN and cognitive impairment is complex and multifaceted. Studies have shown that individuals with mild cognitive impairment (MCI) and AD exhibit deficits in pattern separation [53,54], suggesting that impaired AHN may contribute to cognitive deficits.

#### 2.2.4. Public Health During Lockdown and Environmental Enrichment in Question

This pilot study uses depression symptoms, anxiety behaviours, and cognitive impairment as three proxy measures for AHN and argues that the three measures may mediate any potential relationship between the residential environment and AHN. The contribution of this approach can add a new layer to the impact of lockdown on brain health. The COVID-19 pandemic inadvertently provided an opportunity to examine the impact of housing on human mental well-being, which gives us implicit insights into the prospective impact on AHN as well. We propose that the human house (H) is a macrocosm of the rodent’s impoverished cage, while the city (C) represents a more complex cage. Lockdowns, quarantines, and stay-at-home orders decreased spatial complexity (H − C), followed by a subsequent increase (H + C) as restrictions eased. During that process, mental distress was common in many contexts, raising the question of whether the inhabited environment played a vital role in the experience of mental distress. For instance, large-sample studies, such as the UK Household Longitudinal Study (UKHLS), offered valuable insights into the psychological impact of lockdowns on anxiety and depression before, during, and after the lockdown periods. The UKHLS is a nationally representative longitudinal study tracking the mental health of adults in the UK since 2009. During the pandemic, the study conducted several COVID-19-focused surveys, providing a unique opportunity to examine the mental health consequences of lockdowns. Several studies revealed an increase in distress, anxiety, and depression symptoms during lockdown and stay-at-home orders [17,19,20]. The same problem was observed in Germany [15], Italy [18], the USA [16], and more contexts that we can mention. Those studies suggest that the extent to which the home is an enriched environment may, therefore, be in question.

### 2.3. Housing, Environmental Enrichment, and AHN: From Rodents to Humans

In this section, we exclude the social-related and pandemic-related causes of mental health problems and focus on how physical environmental enrichment can be a variable that leads to an increase or decrease in AHN and its public health proxy measures. However, this section will rely on both rodent and human studies alike due to the abundance of studies on the former, the scarcity of studies on the latter, and the similarity between both subjects reported by [8] to be similar rather than different.

One of the earliest reviews by Olsson et al. [55] provided a comprehensive review of 40 studies published between 1987 and 2000 that investigated the effects of environmental enrichment on the welfare of laboratory mice. The authors discussed the limitations of standard laboratory housing, while environmental enrichment, such as the provision of nesting material, shelters, and climbing structures, aims to improve the housing environment and promote natural behaviours. The review highlighted that mice show strong preferences and motivation to access nesting material and use it to build nests. They also prefer and will work to access more complex cages with shelter and platforms compared to standard cages. Providing nesting material and increasing cage complexity was found to have beneficial effects on mouse behaviour, physiology, and ability to cope with stressors, suggesting improved welfare. We can contrast several pieces of information in that review regarding how the human housing environment can be limited in enrichment—single-storey, little space, limited variability, monotonous surface finishes, limited daylight/sunlight variability, constant temperatures, and similar monotonous design features.

A plethora of studies have been conducted on rodents since then, but few have highlighted the translation of physical environmental enrichment research from rodents to humans. On the one hand, Kempermann et al. [21] argues that the paradigm is relevant to understanding the human brain. The effects of environmental enrichment may provide insights into the mechanisms through which life experiences shape brain function and behaviour. As large cohort studies in humans search for biological markers of successful ageing, animal studies using the environmental enrichment paradigm could help unravel the underlying mechanistic complexity. This approach could involve longitudinal studies that capture the trajectories of brain plasticity in response to environmental factors, as well as multivariate analyses that integrate data across scales from molecules to behaviour. On the other hand, Rojas-Carvajal et al. [24] discussed the potential of using housing condition models in rodents to study the stress associated with modern urban lifestyles in humans. The authors highlight the commonalities between human and animal environmental enrichment and propose adapting these models to investigate the cumulative impact of chronic, low-intensity stressors that are characteristic of urban living. The authors critique current preclinical stress models in rodents, arguing that they often employ excessively severe and unnatural stressors (e.g., electric shocks, immobilisation, and tail pinch) and vary randomly in frequency and intensity to allegedly resemble the stress of modern human life. While these protocols reliably induce stress responses and pathological phenotypes, they may not accurately reflect the stressors experienced by rodents or humans in more natural environments. They propose that rodent models of housing conditions, such as social isolation, standard housing, and environmental enrichment, could be adapted to study the stress of modern urban lifestyles. Most recently, a review by Khalil et al. [22] compares how a non-monotonous interior environment for rodents provides an additive increase to AHN independently from the presence of running wheels or the increase of navigational complexity, revealing that changing complexity, both with and without physical activity, can effectively stimulate neurogenesis. Translating findings from rodent studies to human contexts is a complex endeavour, and probably not straightforward, but it holds great promise for understanding the impact of spatial complexity on AHN. By examining the effects of various environmental enrichment variables on AHN, we may gain valuable insights into the potential benefits and limitations of housing on neuroplasticity, mental health, and well-being.

Nonetheless, not only environmental enrichment but also lifestyles are discussed as important factors to consider towards improving human healthy life expectancy in the context of increased global longevity [56], which is in line with the argument raised in this paper. The interest in the impact of housing and the built environment on the mental health and cognitive function proxies we introduced earlier started gaining attention. A recent study by McCormick et al. [14] reported that homebodies have significantly poorer cognitive function than venturers outside the house and this relationship was not mediated by the number of unique destinations or breadth of community participation activities, suggesting that spending extensive amounts of time in an unchanging environment can be a factor that leads to poor cognitive function, and that this is a potential area for intervention. In this pilot study, we explore the extent to which housing, and associated lifestyle variables, can be perceived as enriched and explore the variables that may contribute to this, if any.

Physical activity is an important lifestyle variable to consider, and it is in line with the recent framework of environmental affordance for physical activity that has been introduced recently as a potential promoter for neurosustainability [57]. It is essential to understand that the effect of walking, cycling, or other forms of mobility that induce physical activity can hardly be isolated from the other effects of increased physical activity. For example, Shin, et al. [58] reported that geospatial complexity has positive effects on people diagnosed with Alzheimer’s disease, and earlier studies have revealed that London taxi drivers had changes in their hippocampus due to navigation, but physical activity in that case was not present as a variable [59]. On the other hand, walking in the form of leisure-time physical, functional, or social activity had a positive effect on the hippocampus, while navigation was not a variable in that case [60]. Hence, we suggest that each variable may have an independent effect, but separating them is often difficult in studies that take place in real-world environments. We can take the example of studies in the lab using a treadmill to test the effect of virtual spatial exploration [61], or studies testing the sequential effect of physical activity and spatial learning [62]. Thus, it was essential to consider the mediating role and potentially confounding effect of lifestyles.

The impact of physical activity on AHN and the hippocampus is well-established. Numerous studies have demonstrated that physical exercise can stimulate AHN in animal models [63,64,65]. Some of the studies highlighted the additive effect of physical exercise, which was also supported by the systematic review by Khalil et al. [22], distinguishing the effect of spatial complexity, change, and physical activity. In humans, Erickson et al. [66] used magnetic resonance imaging (MRI) to examine the effects of aerobic exercise on hippocampal volume in older adults. The researchers found that a one-year aerobic exercise intervention led to a significant increase in hippocampal volume, which was associated with improvements in spatial memory performance. This study provided the first evidence that exercise can modulate hippocampal volume in humans, suggesting a potential role for AHN in mediating these effects and evidencing an increase in BDNF levels. Subsequent research has further supported the link between physical exercise and AHN in humans. A systematic review and meta-analysis by Firth et al. [67] found that aerobic exercise interventions were associated with significant increases in hippocampal volume across a range of age groups. The authors concluded that exercise-induced changes in hippocampal volume might be related to the stimulation of AHN, although direct evidence was still lacking. Most recently, Dadkhah et al. [68] explored experimental and clinical evidence of physical exercise on BDNF and cognitive function in their comprehensive review, supporting the role of physical activity as well.

Furthermore, a growing body of evidence suggests that the intensity and type of exercise may modulate the effects on AHN. High-intensity interval training (HIIT) has been shown to be particularly effective in stimulating neurogenesis and improving cognitive function in animal models [69]. In humans, even a 20-min low-to-medium intensity gardening activity performed by adults was sufficient to increase BDNF and platelet-derived growth factor (PDGF) levels among the elderly participants [70]. If we translate that to how the built environment can embrace or influence physical activity, Khalil [2] suggests walking and step count through the built environment can be a predictor, while Khalil [57] suggests that stairs may be a form of resistance training or high-intensity interval training afforded by the environment. We support this assumption based on a study on humans, proving that a greater amount, duration, and frequency of total daily walking activity is associated with larger hippocampal volume [60]. This relationship was further supported by a recent study showing that free-living physical activity, measured using a wearable device, is associated with larger hippocampus volume as well as greater functional connectivity in healthy older adults [71]. Those studies provide evidence that can help explore the impact of the physical environment on AHN through the proxy measures identified.

## 3. Materials and Methods

### 3.1. Research Design, Hypotheses, and Survey Design

**Figure 1:**
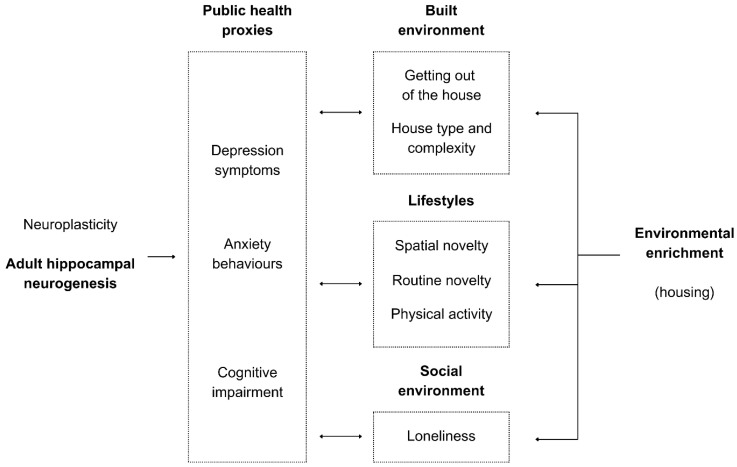
Pilot study design. Depression, anxiety, and cognitive impairments are public health proxies representing changes in adult hippocampal neurogenesis (AHN), while the environmental enrichment of housing is explained through the built environment, lifestyles, and the social environment with an emphasis on perceived loneliness.

The relationship between housing environmental enrichment and AHN is complex, so the design of this pilot study, illustrated in Figure 1, breaks down environmental enrichment into distinct variables and uses proxy measures of AHN.

To measure each of the three public health proxy measures of AHN, three established inventories were used: the Patient Health Questionnaire (PHQ-8) [72,73], the General Anxiety Disorder Questionnaire (GAD-7) [74], and the Cognitive Function Instrument (CFI) [75]. For the PHQ-8 and GAD-7 inventories, participants are usually asked about their experience over the past two weeks on the items provided by each questionnaire. However, for this pilot study, we extended the duration to four weeks to better capture the impact of the various housing environmental enrichment variables on the proxy measures. The CFI, however, asks participants to give their scores to the items compared to their experience a year ago, which we thought may pose a limitation if participants have been living in the same house for one year, leading us to add a question to capture this variability. Participants were asked, “How long have you been living in your current house?”. Those dependent variables are used to explore the potential of one or more independent variables to have a significant effect on their variance. The exact dependencies are discussed subsequently.

#### 3.1.1. Exploring the Effect of the House Compared to Getting Out of the House

The first part of this pilot study aims to explore the actual effect of the house itself on the public health proxy measures of AHN before exploring the nuanced differences in the physical environment of the house or any associated lifestyles. This is achieved by exploring the difference between spending more time in the house (represented by homebodies) and spending more time outside the house (represented by venturers). Hence, hypothesis 1 is formulated as follows:

**Null hypothesis 1** **(H_0_1):** *There is no significant difference in AHN’s public health proxy measures between individuals with high tendencies to leave the house (ventures) and those with low tendencies to leave the house (homebodies).*

The following question was designed for this pilot study to test the first null hypothesis: “How would you describe yourself in terms of your tendency to spend time at home or venture outside your home over the past 4 weeks?”. The following 5-point answers were provided in a quantified manner (1 = Strong homebody, e.g., at home for 19 to 24 h, and outside for 0 to 5 h; 2 = Moderate homebody, e.g., at home for 15 to 18 h, outside for 6 to 9 h; 3 = Neutral, e.g., at home for 10 to 14 h, outside for 10 to 14 h; 4 = Moderate venturer, e.g., at home for 5 to 9 h, outside for 15 to 19 h; 5 = Strong venturer, e.g., at home for 0 to 5 h, outside for 19 to 24 h).

It is important to note that if leaving the house proves to be better than staying in the house, it would be out of the scope of this pilot study to explore variables outside the house that may contribute to the variability of the used public health proxy measures of AHN, which poses a limitation to the scope of this study as it expands to explore the nuanced differences at the scale of the house itself. A recent study has primarily focused on the experience outside the house using GPS tracking [14], which makes both studies complementary rather than posing a limitation to our pilot study.

#### 3.1.2. Exploring the Effect of the House’s Layout Complexity

The second part of this pilot study aims to assess the impact of different house types that represent different layout complexities. This is achieved by developing the following null hypothesis:

**Null hypothesis 2** **(H_0_2):** *There is no significant difference in AHN’s public health proxy measures between different house types with varying levels of layout complexity.*

To test the second null hypothesis, the following question was developed and presented in the beginning: “What type of home do you live in?”. We initially aimed for an equal 20% participant distribution per each of the following categories: Studio flat (self-contained open space), Flat/Apartment (separate rooms on one floor), Terraced house (house in a row of houses), Semi-detached house (one-side shared with neighbour), and Detached house (standalone house). This categorisation assumes that the Studio flat is the least complex and the Detached house is the most complex (generally) since the former has the fewest rooms and floors while the latter has two or more floors, more rooms, and an outdoor space. However, the terraced, semi-detached, and detached house typologies contrast the studio flat and apartment typologies, as the three former typologies have more than one floor while the latter typologies have only a single storey.

Additionally, another question was developed to add more depth to the anticipated results by exploring whether the separation of activities in separate spaces (regardless of the architectural layout complex) or their overlap (as a way of zoning) has an effect: “How often do you use the same area/room for multiple activities over the past 4 weeks at your house?”. The answers were: 1 = Never (e.g., I use separate areas for sleeping, working, eating, and leisure activities, like resting on a chair, eating on a table… etc.), 2 = Rarely (e.g., I use separate areas, but might occasionally use the same area for two activities), 3 = Often (e.g., I use the same area for a few activities, but use separate areas for one or two activities), or 4 = Always (e.g., I use the same area for all activities, like sleeping, working, eating, and leisure; for example, resting, eating, and relaxing all on a sofa). In other words, this question assesses experienced complexity from complexity that is spatial.

#### 3.1.3. Exploring the Effect of Experiencing Novelty and Change in the House

The third part of this pilot study aims to assess the impact of experiencing novelty and change as either a lifestyle- or spatially-driven variable, and that is achieved by developing the following null hypothesis:

**Null hypothesis 3** **(H_0_3):** *There is no significant difference in AHN’s public health proxy measures between individuals with low tendencies of change experienced in the built environment and those with high tendencies.*

Three questions were developed to test the third null hypothesis from three perspectives: the spatially-dependent change of environment, lifestyle-based routine novelty, and change or novelty experienced through changing an interior setting.

The first question was: “How often did you change where you are used to doing each activity over the past 4 weeks at your house?”. The answers were: 1 = Never (e.g., I never change where I do each activity), 2 = Rarely (e.g., I rarely change where I do each activity, but I might try a new area occasionally), 3 = Often (e.g., I often change where I do each activity, but I have some preferred areas), or 4 = Always (e.g., I always change where I do each activity and rarely use the same area twice).

The second question was: “How often did you change your routine over the past 4 weeks at your house?”. The answers were: 1 = Never (e.g., I follow the same routine every day), 2 = Rarely (e.g., I have a similar routine with minor variations), 3 = Often (e.g., I have a mix of routine and new activities), or 4 = Always (e.g., I do completely new things every day). 

The third question was: “Over the past 2 months, how often have you relocated, changed, or added an item or piece of furniture in your house?”. The answers were: 1 = Never (e.g., I have not made any changes to the location or arrangement of items or furniture), 2 = Rarely (e.g., I made a few minor changes, like moving a small item or two), 3 = Often (e.g., I made several changes, like rearranging furniture or adding new items), 4 = Always (e.g., I made significant changes, like completely reorganising rooms or adding multiple new pieces of furniture).

#### 3.1.4. Exploring the Effect of Physical Activity Through the House

The fourth part of this pilot study aims to assess the impact of various levels of physical activity experienced throughout the house. This is achieved by developing the following null hypothesis:

**Null hypothesis 4** **(H_0_4):** *There is no significant difference in AHN’s public health proxy measures between individuals with low and high tendencies of physical activity at their home.*

The following question was developed to test the fourth null hypothesis: “Over the past 4 weeks at your house, were you mostly..”. The answers were: 1 = Sedentary (sitting, lying down, or engaged in activities with minimal movement), 2 = Lightly active (standing, light housework, or activities with occasional walking), 3 = Moderately active (regular housework, gardening, or activities with frequent walking), or 4 = Very active (physically demanding housework, exercise, or activities with continuous movement).

#### 3.1.5. Comparing the Effect of Physical Enrichment with Social Enrichment

Last but not least, the fifth null hypothesis aims to compare the effect of both physical enrichment and social enrichment firstly through developing the most significant model fit of physical enrichment based on testing the first four previous hypotheses and then comparing the combined significant effect with that of social enrichment. Perceived loneliness was used as an indicator of social enrichment rather than the actual presence of others, as the former is evidenced to be associated with smaller brain volumes [76]. Hence, the following question was developed: “At your house over the past 4 weeks, how often have you felt lonely?”. The answers were: 1 = Never, 2 = Rarely, 3 = Sometimes, 4 = Often, or 5 = Very Often). The fifth null hypothesis, therefore, was as follows:

**Null hypothesis 5** **(H_0_5):** *The percentage of variance in AHN’s public health proxy measures explained by physical enrichment and social enrichment is not dramatically different.*

### 3.2. Data Analysis

Three statistical methods—linear regression, one-way ANOVA, and Pearson correlation—were employed to test the five hypotheses, identify effect sizes, and explore confounding variables. Additionally, we conducted Bonferroni post hoc tests where appropriate. All data analysis was conducted using the SPSS software version 29.0.1.0 (171).

### 3.3. Power Analysis and Sample Size

Power analysis was done separately using SPSS for each statistical method to determine the minimum sample size. Since no prior studies exist, the sample size was calculated three times based on the respective small, medium, and large effect sizes listed by Serdar et al. [77] for each statistical method, respectively. This approach is thought to be helpful in determining the prospective limitations that may arise with any observed significance. The *p*-value and the statistical power value are 0.05 and 0.8, respectively.

For single and multiple regression, effect size values of 0.02, 0.13, and 0.26 represent small, medium, and large effect sizes, respectively. On the one hand, if using one independent variable, the sample size must be 395, 63, and 33 participants if the effect sizes are small, medium and large, respectively. On the other hand, if using a maximum of five independent variables (based on the developed hypotheses) that may potentially combine in one multiple-regression model, the sample size must be 647, 105, and 56 participants if the effect sizes are small, medium and large, respectively.

For the one-way ANOVAs, Eta Square (η^2^) effect size values of 0.01, 0.06, and 0.14 represent small, medium, and large effect sizes, respectively. If comparing five house types with expected equal weights, the sample size must be 1190, 195, and 80 participants if the effect sizes are small, medium and large, respectively.

For the Pearson correlation analysis, effect size values of 0.2, 0.5, and 0.8 represent small, medium, and large effect sizes, respectively. Hence, the sample size must be 193, 29, and 9 participants if the effect sizes are small, medium and large, respectively.

This pilot study’s sample size of 142 participants is considered sufficient only for variables with regression analyses’ medium and large effect sizes, ANOVAs’ large effect size, and Pearson correlation coefficients with a medium to large effect size only. Variables with smaller effect sizes, if any, will need more research or to be interpreted with the sample size limitation.

### 3.4. Distribution

Ethical approval was obtained from the authors’ institution for this study, which aimed to anonymously collect different residential information from participants and through which part of that complex survey is presented in this pilot study. Afterwards, the survey was distributed online using a survey administration platform, where participants were compensated for completing the survey. The survey was distributed to participants with no involvement from the researcher except for designing the survey and setting the eligibility criteria for participation: limited to participants above 18 years of age, living in England, UK. Participants had to consent to participation based on the provided introduction and terms before participating in the survey. After completing the survey, participants were compensated for completing the survey by the researcher but anonymously through the survey administration platform without revealing the identity of participants to the researcher, who receives responses anonymously referenced with IDs. The platform automatically provides the researcher with each ID’s demographic information, such as city, age, gender, economic status, and marital status. This provides the researcher with more insights into the sample while maintaining anonymity.

## 4. Results

The 142 participants who successfully completed this online survey were all from England. The sample characteristics were 56.3% females and 43.7% males, Mage = 42.54 with a std. deviation of 14.834 and a range of 60 (18 to 78 years old).

We report that the sample consisted of self-reported mentally healthy participants who experience overall low depression symptoms, anxiety behaviours, and cognitive impairment. Firstly, regarding depression symptoms, which were scored across eight items on a scale from 1 (least frequent) to 4 (most frequent), the sample had a mean value of 1.79 with a std. deviation of 0.71. Secondly, regarding anxiety behaviours, also scored across seven items on a scale from 1 (least frequent) to 4 (most frequent), the sample had a mean value of 1.65 and a std. deviation of 0.74. Lastly, regarding cognitive impairment, participants’ scores were calculated as the sum of scores based on their yes/no/maybe answers, representing scores of 1, 0, and 0.05, respectively, to the fourteen items based on their cognitive experience compared to one year ago, showing that the sample had a mean value of 2.9 with a std. deviation of 3 and a range of 12. Additionally, 85.9% of the participants had been living in their house for over one year, which reduces the limitation of how the CFI enquires about long-term effects that we used to test the impact of only one place of residence. Reliability analysis was conducted for the items of PHQ, GAD, and CFI separately, and found that the Cronbach’s Alpha for GAD’s seven items was 0.934, while for PHQ’s eight items, it was 0.892, and for CFI it was 0.848. As all are above 0.7 and closer to 1, this suggests reliability.

This pilot explored the potential contribution of population characteristics such as income and age in explaining the potential variance of depression, anxiety, or cognitive function. We found a statistically significant relationship between all variables when explored separately. On the one hand, income had a significant relationship with depression (*p*-value ≤ 0.001; B = 1.988; R square = 0.024), anxiety (*p*-value ≤ 0.001; B = 1.690; R square = 0.001), and cognitive function (*p*-value ≤ 0.001; B = 3.404; R square = 0.007). On the other hand, age had a significant relationship with depression (*p*-value ≤ 0.001; B = 1.935; R square = 0.005), anxiety (*p*-value ≤ 0.001; B = 1.990; R square = 0.026), and cognitive function (*p*-value ≤ 0.001; B = 3.293; R square = 0.002). However, it is important to note that the observed effect sizes are almost negligible as the R square values ranged between 0.001 and 0.026.

### 4.1. The Effect of Housing Physical Enrichment on AHN’s Public Health Proxies

#### 4.1.1. The Effect of the House Compared to Getting Out of the House

Overall, the sample consisted of 61.3% strong to moderate homebodies, while only 10.6% of participants were moderate to strong venturers, and 28.2% were neutral. This sample, therefore, predominantly represented homebodies, helping explore the nuanced differences at the scale of the house.

Regression analysis was conducted between the tendency to leave the house and each of the three proxy measures of AHN (depression, anxiety, and cognitive impairment). We found a very strong statistically significant inverse relationship between high tendencies to leave the house and depression symptoms (*p*-value ≤ 0.001; B = −0.295; R Sq. = 0.171), anxiety behaviours (*p*-value ≤ 0.001; B = −0.217; R Sq. = 0.085), and cognitive impairment (*p*-value = 0.004; B = −0.727; R Sq. = 0.058). In other words, through the regression method, high tendencies to spend more time in the house were found to explain 17.1% of the reported depression symptoms, 8.5% of the anxiety behaviours, and 5.8% of the cognitive impairment. Before interpreting the effect size, according to Cohen et al. [78], this is considered a low-to-moderate effect size. It is worth noting that the sample is mentally healthy, and those results are relative to non-severe depression, anxiety, or cognitive impairment. The effect size through regression analysis should be treated cautiously until future research with a large sample size takes place and uses a numerical continuous scale of time spent outside the house and at home.

The effect sizes identified above through the regression analysis were based on the assumption that the difference between a strong homebody and a strong venturer is only subject to the continuous scale, but each score encompasses a range. Thus, this study used an additional statistical test, the one-way ANOVA, to test the variability between the five groups where the Likert scale is treated as categorical rather than continuous.

The first one-way ANOVA was conducted using the independent variable as the factor and the CFI mean values as the dependent variable, yielding a significant moderate-to-large partial eta squared effect size (η^2^ = 0.114; *p*-value = 0.002) as shown in Table 1. Similarly, McCormick et al. [14] used independent *t*-tests and found a significant medium effect size of d = 0.68. This is in line with our findings and, therefore, we suggest that the one-way ANOVA is a more effective statistical tool than regression in that regard. Our study’s sample (*n* = 142) was skewed over 61% towards homebodies, while McCormick et al.’s [14] sample (*n* = 97) had 23 homebodies and 74 ventures. Hence, this study confirms that getting out of the house is beneficial for improving cognition, and our subsequent findings can provide more insights.

Subsequently, further separate one-way ANOVA tests were conducted with depression and anxiety as the dependent variables to get more insights into the potential impact on AHN holistically. The results are also presented in Table 1. For depression, the analysis revealed a significantly large effect size (η^2^ = 0.205; *p*-value < 0.001). This substantial effect size suggests that approximately 20.5% of the variance in depression scores can be attributed to group differences. The analysis of anxiety also yielded significant results (*p*-value = 0.003), but with a medium-to-large effect size (η^2^ = 0.107) that needs to be interpreted cautiously given the sample size, representing 10.7% of the variance in anxiety.

Therefore, based on the power analysis conducted earlier, our sample size, and the calculated effect size, we cannot accept null hypothesis 1 that there is no significant difference between the tendencies to leave the house and the proxy measures of AHN. This finding is in line with [14], who state that spending more time outside the house is better for cognitive function. The relationship between venturing outside the house and improved cognition is not mediated by the number of unique destinations visited or the breadth of community participation, suggesting that it can be due to spending time in an unchanging environment [14]. This may be in line with a recent review that discusses the role of changing spatial complexity in rodents’ housing and the potential translation to human contexts as it promotes AHN [22]. We suggest that it might be due to navigational complexity experienced at the urban scale [58], physical activity through increased walking tendencies [60,71], both, or in combination with other unexplored variables.

#### 4.1.2. The Effect of the House Type’s Complexity

The premise of this study is that the various complexities of the house, represented by different house types that participants can easily align with, can help explore if any variables contribute to potentially nuanced differences in any of the proxy measures of AHN.

Regarding the percentage of participants in each house type, 9.9% of participants live in a studio flat, 23.2% in an apartment flat, 19.7% in a terraced house, 22.5% in a semi-detached house, and 24.6% in a detached house. Though it was expected to have equal or close percentages, there was a lack of sufficient numbers of participants living in studio flats, which posed a limitation to this house type and its open-plan form of complexity.

Firstly, we conducted correlation analyses and found a weak positive correlation between house type (five types) and income level (*p*-value = 0.014, Pearson *r* = 0.286). We also observed a weak positive correlation between the subjective perception of spaciousness to houses similar in type and the respondent’s house type in the five groups (*p*-value = 0.014, Pearson *r* = 0.206). In other words, detached houses tend to be perceived as more spacious than studios in general, but this correlation is very weak.

A series of one-way ANOVA tests were conducted to examine the effects of the house type (five types) on depression, anxiety, and cognitive impairment scores. The analyses revealed varying levels of association between housing type and these proxy measures of AHN. The first ANOVA test showed a statistically significant variance in depression (*p*-value = 0.017, η^2^ = 0.084) which was explained by the house type, as shown in Table 2. The eta-squared value of 0.084 suggests a medium effect size, with housing type accounting for 8.4% of the variance in depression scores. Post-hoc comparisons using the Bonferroni correction indicated that residents of Flats/Apartments (M = 2.1436, SD = 0.78626) reported significantly higher depression scores compared to those living in Semi-Detached Houses (M = 1.6472, SD = 0.60035, *p*-value = 0.043) and Detached Houses (M = 1.6463, SD = 0.69226, *p*-value = 0.035). The mean differences were 0.49645 and 0.49735, respectively. The lack of a significant relationship with the terraced house could be due to its relatively smaller terraced house type sample size, unlike the apartment, semi-detached, and detached houses, which have equal percentages of participants. This limitation also applies to the dramatically low studio-type sample size.

**Figure 2:**
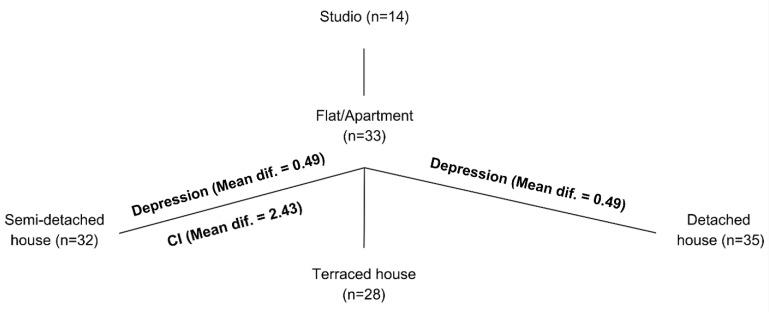
Significant mean difference between house types.

The difference between the five house types did not explain the significant variance in anxiety (*p*-value = 0.107, η^2^ = 0.054), as shown in Table 2. Despite the lack of statistical significance, descriptive statistics showed that Flat/Apartment residents reported the highest mean anxiety scores (M = 1.9594, SD = 0.94792), while Semi-Detached House residents reported the lowest (M = 1.5391, SD = 0.60116). The difference between the five house types, however, explains the significant variance in cognitive impairment (*p*-value = 0.007, η^2^ = 0.096), as shown in Table 2. The eta-squared value of 0.096 suggests a medium effect size. Post-hoc comparisons using the Bonferroni correction indicated that residents of Flats/Apartments (M = 4.2576, SD = 3.64032) reported significantly higher cognitive impairment scores compared to those living in Semi-Detached Houses (M = 1.8281, SD = 2.06198, *p*-value = 0.009), with a mean difference of 2.42945. No other pairwise comparisons yielded statistically significant results. Figure 2 illustrates the significant values observed through the post-hoc comparisons.

This pilot study excluded the studio house type and conducted the ANOVA tests again to see if this may help better explore the variances between the remaining four house types, with relatively closer frequencies ranging between 28 and 35 participants each. However, the results are not much different where the impact of the house types on the variance in depression subtly increased in significance and effect size (*p*-value = 0.007, η^2^ = 0.093), and also with cognitive impairment (*p*-value = 0.006, η^2^ = 0.096).

It is difficult at this point to reject or accept null hypothesis 2 due to the limited sample size compared to observed effect sizes and before exploring the potential role played by other variables. However, we have observed some significant relationships with medium to large effect sizes that at least suggest not accepting null hypothesis 2 that the difference in house type complexity does not explain any difference in AHN’s public health proxy measures.

Lastly, regression analysis was conducted between the tendency to use a separate space per activity and each of the AHN proxy measures (depression, anxiety, and cognitive impairment). However, we did not find any significance with depression (*p*-value = 0.530), anxiety (*p*-value = 0.230), or cognitive impairment (*p*-value = 0.129). Therefore, the house type complexity and the use of that complexity in a separate, non-overlapping manner are two separate topics.

### 4.2. The Effect of Residential Lifestyles on AHN’s Public Health Proxies

#### 4.2.1. The Effect of Experiencing Change and Novelty in the House

To test hypothesis 3, a regression analysis was conducted between each of the three suggested independent variables (the tendency to change the space used for each activity, the tendency to change routine, and the tendency to change the interior setting) with each of the AHN’s public health proxy measures (depression, anxiety, and cognitive impairment).

As shown in Table 3, firstly, we found a significant linear relationship between changing the space used for each activity and depression (*p*-value = 0.003) and anxiety (*p*-value = 0.020), but not with cognitive impairment (*p*-value = 0.122). Secondly, we found only a significant linear relationship between changing routine and anxiety (*p*-value = 0.014). This might indicate that change and novelty, in their current forms explored, can reduce anxiety, as evidenced in rodents [22]. Thirdly, we tested if changes made to the interior setting of the house over the past two months, if any, yielded any difference in depression, anxiety, or cognition. We observed no statistically significant significance with depression (*p*-value = 0.656), anxiety (*p*-value = 0.324), or cognitive impairment (*p*-value = 0.321), suggesting that the direct translation of rodent-based changes in interior spatial complexity into a human context [22], without objective quantification first, needs to be carried out through further exploration.

This section concludes that change/novelty may be a key factor as evidenced among rodents and noted to be associated with reduced anxiety despite the limitation of sample size and effect size compatibility. Therefore, we cannot accept or reject null hypothesis 3 at this point until more research takes place. We conducted a correlation analysis to guide future research in that regard. There is a significant correlation between the two independent variables: changing the space used for each activity and changing the routine (*p*-value ≤ 0.001; Pearson *r* = 0.322), and a weak positive correlation between the tendency to change the interior setting and changing the space used for each activity (*p*-value = 0.022; Pearson *r* = 0.192), and changing the routine (*p*-value = 0.012; Pearson *r* = 0.210). This should be taken into consideration in future research exploring how novelty experienced in or through the housing environment can be translated from rodents to humans.

#### 4.2.2. The Effect of Physical Activity Throughout the House

To test null hypothesis 4, regression analysis was first done between the tendency to be physically active in the house and each of AHN’s proxy measures. As shown in Table 4, we found a very strongly significant inverse relationship between the high tendencies of physical activity in and throughout the house with depression (*p*-value = 0.001), no significant relationship with anxiety (*p*-value = 0.278), and a significant relationship with cognitive impairment (*p*-value = 0.040). However, the significant effect sizes were small, which requires future research with a larger sample size.

Hence, we cannot reject null hypothesis 4, but more research is needed to formulate an alternative hypothesis. The novel framework about environmental affordance for physical activity can find those insights helpful [57]. In that regard, and to provide further insights that can help future research, we conducted a one-way ANOVA test between the tendency to perform physical activity and the house type. At first, we found no significant relationship when exploring the variance in physical activity based on the difference between the five house types (*p*-value = 0.055), but when we excluded the studio house type due to not meeting the sufficient sampling saturation then conducted another one-way ANOVA test between the remaining four house types and the tendency to perform physical activity, we found a significant relationship (*p*-value = 0.034). Interestingly, when excluding the apartment house type, we found no statistically significant variance in physical activity between the terraced, semi-detached, and detached house types (*p*-value = 0.95), which are all multi-storey houses. Table 5 presents the results of the three analyses. This suggests that the studio house type’s sample size poses a limitation. In contrast, the variance explored in combination with the apartment may suggest that the presence of a staircase is what may induce a variance in physical activity as suggested to induce affordance for physical activity [57].

Further, we conducted correlation analyses between the tendency to be physically active in the house and the house type (five types), finding a significant weak positive correlation between the two variables (*p*-value = 0.014; r = 0.206). Further correlational analyses were conducted between the spatial lifestyle-related variables. There were weak to moderate positive correlations between the tendency to be physically active in the house and each of the tendencies to leave the house (*p*-value ≤ 0.001; Pearson *r* = 0.368), to change the space used for each activity (*p*-value ≤ 0.001; Pearson *r* = 0.331), and to change the routine (*p*-value = 0.004; Pearson *r* = 0.238). The correlations between those variables are summarised in Table 6.

At this point, it is difficult to reject or accept null hypothesis 4 due to the medium to large effect size, but it appears to have a significant positive relationship with the public health proxy measures of AHN, probably due to the provision of stairs in multi-storey houses.

### 4.3. Comparing the Effects of Physical Enrichment and Social Enrichment

Before comparing the effects of physical enrichment with social enrichment, we present the results of multiple regression analyses conducted to explore the most significant model.

Firstly, a multiple regression analysis was conducted with depression as the dependent variable, and interestingly, a holistically significant model was only found when including the single-storey flat/apartment with the three types of multi-storey houses (a total of four house types). We confirm this assumption since no consistency of significance within the model was found when comparing only the variance between the three house types (a total of three house types) with the presence of the physical activity variable, as shown in Table 7. 

The model fit significance and R square values are presented in Table 8, where it is evident that the effect size of house type and physical activity combined with depression explained 13.4% of the variance. It is important to note that this percentage is significantly high since the regression model conducted earlier between the house type variance (four house types) and depression resulted in an R square value of 0.061 (6.1%).

We further conducted multiple-regression analyses and found, also shown in Table 8, that the dependency of depression on the house type (four types), physical activity, and change of space used for each activity combined increased to explain 16.5% of the variance. This means that an additional 3.1% of the variance in depression is explained by the change of space used for each activity, which might be facilitated by the variance between single-storey and multi-storey houses, which is an interesting area of research that needs to be further investigated.

Given that the model fit number 5 and also number 1 in Table 6 each have a large effect size larger than 0.13, we strongly accept both models since the sample size of this paper is sufficient for their effect size.

**Figure 3:**
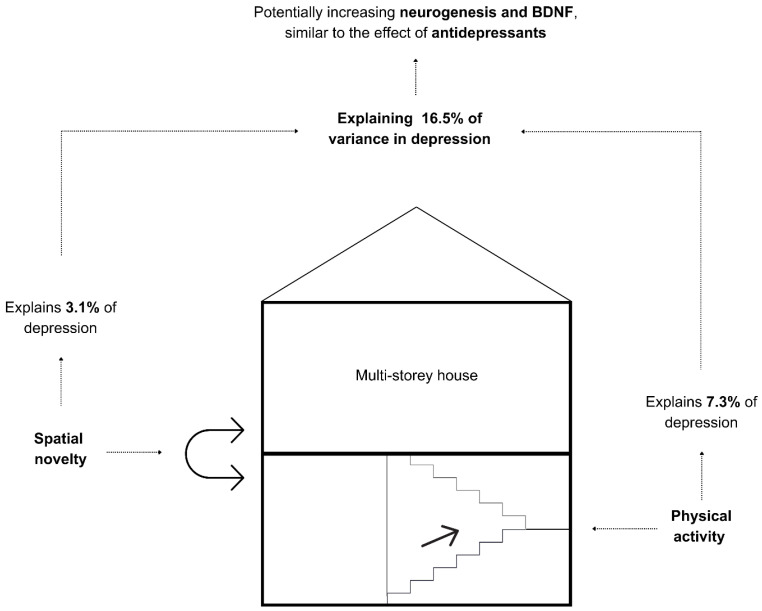
Variance in depression (proxy of adult hippocampal neurogenesis) explained by the variability of housing structural enrichment. The different house types combined with the effect of physical activity through the house and the effect of spatial novelty (changing space used for each activity) together explain 16.5% of the variance in depression. Within this percentage, physical activity throughout the house explains 7.3%, while spatial novelty explains 3.1%, and the independent variable explaining the remaining percentage requires further research.

Taken together, while it was revealed earlier that staying in the house explains 20.5% of the variance in depression compared to getting outside the house, we further report that staying in a single-storey house compared to multi-storey houses explains 16.5% of the variance in depression predominantly through physical activity arguably by the presence of a staircase (7.3%), and the experience of changing the space used for each activity by living in a more complex multi-storey house (3.1%), while an additional yet-to-be understood characteristic of living in a multi-storey house explains the remaining variance (6.1%). This complex relationship is illustrated in Figure 3.

Last but not least, to test null hypothesis 5, a regression analysis was conducted first between perceived loneliness and each of the three AHN proxy measures. We found a statistically significant relationship between loneliness and depression (*p*-value ≤ 0.001; B = 0.327; R Sq. = 0.266), anxiety (*p*-value ≤ 0.001; B = 0.399; R Sq. = 0.361), and cognitive impairment (*p*-value ≤ 0.001; B = 0.878; R Sq. = 0.107). We report that loneliness had a noticeably large effect size on the three proxy measures of AHN.

**Figure 4:**
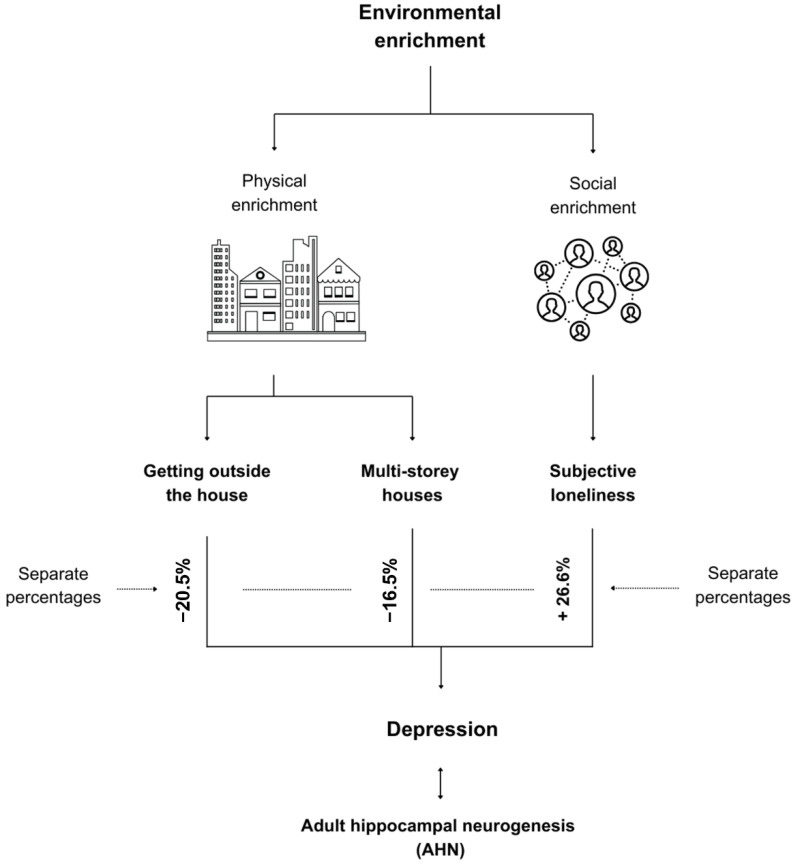
Percentage of variance in depression through physical enrichment and social environments potentially regulating AHN.

Hence, loneliness explained 26.6% of the increase in depression while getting out of the house explained 20.5% of the reduced depression, and living in a single-storey house compared to living in a multi-storey house explained 16.5% of the variance in depression due to physical activity being potentially dependent on the presence of a staircase, spatial novelty exemplified by changing the space used for each activity, and other characteristics of complexity yet to be understood. Figure 4 illustrates this key conclusion. Hence, social and physical enrichment are equally important, and we cannot reject null hypothesis 5. While insights are predominantly obtained at the architectural level, the sample in this pilot study represents homebodies predominantly, which does not make the limitation of lacking an understanding of the influential variables at the outdoor environment scale critical. However, a study by McCormick et al. [14] provides additional insights into the other perspective.

We may assume that the combined percentage regarding the reported variance in depression is due to the combined adverse effects of physical and social enrichment, but we urge the critical consideration of the combined effect of both percentages due to the potential presence of confounding variables. Future research needs to address each of these problems separately and hopefully in conjunction for greater benefits on public health and neuroplasticity.

## 5. Discussion

The results of this pilot study provide multiple insights regarding potential relationships between attributes of the residential built environment and the public health indicators of AHN, addressing multiple gaps and trends in the existing body of knowledge. While limited in sample size (*n* = 142), this pilot study was adequately powered to detect acceptable effect sizes unless stated otherwise. Future studies building on these findings may benefit from larger sample sizes depending on the research questions and effects under investigation.

Overall, there is a starkly significant difference in the variance of the public health proxy measures of AHN between spending time in the house and spending time outside the house. At this point, it is still unknown whether that is due to the out-of-home environment involving more urban navigation, more exposure to novel experiences, higher tendencies of physical activity, or altogether. Homebodies demonstrated significantly poorer cognitive function than participants who venture more outside their house, noting that this relationship is not mediated either by the number of unique destinations or the breadth of community participation activities, suggesting that spending extensive amounts of time in an unchanging environment can be a crucial factor negatively affecting cognitive function [14]. Walking can mitigate those changes as well since it acts as a form of physical activity [57], while increased urban environmental complexity is selectively linked to larger brain volumes related to allocentric navigation, including the hippocampus, fewer Alzheimer’s disease diagnoses, and stronger spatial behaviour performances [58].

Regarding the house type’s layout complexity, there is a growing interest in the relationship between complex layouts and arousal, fascination, and unusualness [79], and this paper adds a layer to that topic of interest by explaining how this form of layout complexity has an impact on public health and well-being and prospectively may affect AHN. This pilot study has considered five general forms of layout complexity: a studio in the form of an open-plan single-storey house, an apartment in the form of a compartmentalised single-storey house, and terraced, semi-detached, and detached houses as three houses that share a multi-storey layout complexity yet may vary in the presence of the number of rooms. One of the limitations in this pilot study is the need for more sufficient participants living in a studio house typology, which has led to multiple regression analyses to understand the significant differences between the remaining four house types representing layout complexities and the three proxy measures of AHN at certain points. Interestingly, we observed no significant difference between the three multi-storey house types (terraced, semi-detached, and detached) but observed significant differences when including the apartment/flat house type in the comparison.

We observed a potential increase in physical activity arguably and potentially through the provision of a staircase. Thus, we warrant the mere increase of footprint as it is evidenced in rodents to not be sufficient for increasing physical activity as well [80]. We assume that physical activity’s dependency on the house type or layout complexity can be exemplified in the provision of a staircase because we could not find significant differences between the terraced, semi-detached, and detached houses but only found a significant difference when the single-floor apartment was included in the comparison. We also highlight that while such a significant difference was found, other spatial or habitual factors or individual differences driving the use of the staircase may need further exploration. The significance of the presence of stairs is supported by a recent framework explaining how it helps reach metabolic equivalents (METs) needed for hippocampal neurosustainability [57]. This is in line with earlier research by Baker et al. [3] suggesting that a multi-storey house is more likely to increase physical activity than a single-storey house due to the use of the staircase to access various domestic spaces inside the house. The authors stated “Three-storey homes are likely to increase personal physical energy expenditure... Climbing one floor by stairs accounts for 3.3% of extra daily energy expenditure, and getting up to 20 times from a seated position equates to about 10% of a healthy daily total of metabolic activity” [3]. We urge future research to explore the general presence of staircases, nuanced differences between different staircase types (straight, l-shaped, spiral … etc.), and riser heights on influencing various METs, physical activity levels, and potential influences on AHN either by using a biomarker as BDNF or by comparing hippocampus volumes [57]. Our findings in this pilot study are in line with neuro-evidenced research on the interrelationship between depression, AHN, physical exercise, and BDNF [34,81,82,83].

It is suggested to make circulation an enjoyable experience and provide rewards for the movement through good natural light, views, opportunities for spatial variation and encounter, use of artwork, etc. [3]. All those examples expound how future research can experiment with the insights identified in this pilot study. Thus, we suggest future research to explore interior variations encouraging navigation and switching floors [84], but in the housing context. If the house can further provide navigational complexity, this can yield more positive neuroplasticity outcomes. This is important because navigation and space exploration have positive effects on neuroplasticity at the macro scale [61,85]. Thus, further consideration is needed.

We found that changing the space used for each activity and changing the routine has a potential role towards reducing anxiety, as evidenced among rodents [22]. At this preliminary stage, we provide this as concrete evidence that changes in the built environment can be of importance to public health and neuroplasticity, and we further support this through juxtaposing our findings with the suggestions made by McCormick et al. [14] getting outside-the-house results in improved cognition due to changing the environment. However, we found that changing the interior setting with the given assumptions yielded no significant variance in any of the three proxy measures of AHN. Future research needs to experiment with various forms of change in the residential and other built environment types on changes in anxiety behaviours and potentially AHN.

The necessity to take this study further lies in the observed almost equal effect of both physical and social enrichment on depression. We urge future research to experiment with how increasing complexity, change and novelty, and physical activity in and throughout the built environment can enhance well-being and physical activity and how, if combined with forms of co-living [86], can result in more positive neuroplasticity outcomes. This pilot study provides insights into the neuroscientific investigation of singular living [87]. We also urge considering cultural and contextual differences when replicating the findings of this study in a different context. This paper contributes to the vision of exploring the pathway between the physical environment and mental health [1], environmental affordance for physical activity for neurosustainability [57], the design of healthy homes [3], and neurosustainability [2], offering valuable insights into the definition of environmental enrichment.

This pilot study highlights the necessity of both physical activity and spatial novelty in potentially regulating AHN. However, the application of physical activity in the human context appears to be more feasible than experienced novelty despite the significance of both. The pilot study suggests that staircases may have a relationship with physical activity, while spatial novelty may potentially be driven by the presence of more floors aside from the staircase’s use or lack of use We suggest that physical activity, arguably through the presence of stairs, explains the reduction in depression by 13.4%, where this reduction acts as an antidepressant that is evidenced in earlier research to be in a linear relationship with neurogenesis through the neurotrophic factors such as BDNF [88,89]. We advise future research to focus on depression as the most salient proxy of neurogenesis. Most recent work suggests that the level of environmental enrichment is potentially modulating the presence and severity of MDD at a clinical level; however, it can also influence at a neuroplastic level through the promotion or inhibition of BDNF [90], which is in line with our argument in this pilot study.

In conclusion, and despite some observed limitations regarding the sample size and effect sizes found in this pilot study, we summarise our key findings as follows:

1. Compared to perceived loneliness, the physical environment accounts for an equally significant impact on the three public health proxy measures of AHN, particularly depression, urging future research to prioritise both the physical and social environments of the house.
2. While spending more time outdoors is generally associated with better overall public health proxy measures of AHN, future research is advised to explore more additional factors at the urban scale contributing to enhanced AHN, which we suggest are related to spatial complexity, navigation, walking, and changing the environment.
3. The house can compensate for the shortcomings of spending more time at home through the provision of stairs as a means of structural enrichment. Stairs appear to potentially have the capacity to increase physical activity, which holds great promise for both depression and AHN.
4. Future research can benefit from an objectively quantified approach for defining spatial complexity and environmental affordance for physical activity at the level of the house, which was a limitation in this study, relying mostly on subjective evaluation.
5. Spatial novelty and change, while arguably promoting neurogenesis, are more significant in reducing anxiety. Future research may experiment with higher frequencies of changing interior spatial complexity or through other interior or architectural means.
6. Architectural affordances for physical activity and spatial exploration are interesting areas of research that would be of interest to interdisciplinary collaborations.
7. Depression is the most salient proxy measure of AHN, and its variance is most explained by the independent variables found to be significantly influential.
8. Using measures such as BDNF or conducting MRI brain scans for potential tracing of AHN-related changes can provide more confident insights since depression may be confounded by a plethora of variables.
9. While the stimulation of physical activity can be a promoter for reduced depression to increase AHN or reverse its decline, depression can promote physical inactivity, which may inhibit the use of the structurally enriched housing environment. This area of research needs to be further explored.

Future policy planning can benefit from the insights offered by this pilot study where the built environment shows multiple significant correlations with public health proxy measures of neurogenesis in humans, which is a process that persists even until the tenth decade of life, e.g., policy-making concerning ageing [91,92]. However, the insights are applicable to all generations since neurogenesis is a process that is not exclusively associated with a specific stage in life. Furthermore, the impact of student accommodation environments on mental health [93,94,95], which is a very critical topic, can highly benefit from the insights offered in this pilot study. Last but not least, policy-making in response to the reported housing-related mental health conditions, heavily reported during the COVID-19 lockdown [96,97,98,99], can also benefit from the insights offered in this study. While this pilot study has a limited sample size, the topic it covers is becoming increasingly important. Most recently, research talks about linking neuroimaging and mental health to satellite imagery of measurements of macro-environmental factors [100].

## 6. Conclusions

While preliminary, the results of this pilot study offer a plethora of novel insights into potential relationships between attributes of the housing environmental enrichment and the public health proxy measures (depression, anxiety, and cognition) related to AHN. Several thought-provoking findings emerged to call for further rigorous exploration. While the city may be a more enriched form of an environment, the house can compensate through its own unique way of enrichment, arguably through the provision of staircases and encouraging spatial novelty through the provision of multiple floors. This pilot study’s sample size limitation urges cautious interpretation of results reported with low to medium effect size. Future research should focus on specific variables using larger sample sizes, control for household characteristics when examining settings’ independent variables, and use biomarkers and magnetic resonance imaging on variables that have medium to large effect sizes. More research is critically needed to explain further how those proxy measures stand for AHN in humans and to test environmental enrichment variables identified through this study using a wide range of methods.

## Figures and Tables

**Table 1:** Variance in public health proxy measures of AHN by time spent outside the house.

Getting Out of the House ***	ANOVA					ANOVA Effect Sizes		
	Sum of Squares (Between Groups)	df	Mean Square	F	Sig.	Point Estimate (Eta Estimate)	Confidence Interval	
							Lower	Upper
Depression	14.547	4	3.637	8.826	<0.001	0.205 *	0.080	0.300
Cognitive impairment	143.764	4	35.941	4.385	0.002	0.144 *	0.017	0.197
Anxiety	8.352	4	2.088	4.125	0.003	0.107 **	0.014	0.190

* Large effect size that the sample size of this paper strongly supports. ** Medium to large effect size that needs to be interpreted cautiously given the sample size limitation. *** Independent variables potentially influencing the effects are a changing environment [14,22], environmental complexity [58], and walking [57,60,71].

**Table 2:** Variance in public health proxy measures of AHN explained by the house type complexity (five types).

House Type (Five Types) and Complexity	ANOVA					ANOVA Effect Sizes		
	Sum of Squares (Between Groups)	df	Mean Square	F	Sig.	Point Estimate (Eta Estimate) *	Confidence Interval	
							Lower	Upper
Depression	5.953	4	1.488	3.135	0.017	0.084 **	0.003	0.160
Cognitive impairment	121.974	4	30.494	3.650	0.007	0.096 **	0.008	0.176
Anxiety	4.164	4	1.041	1.939	0.107	0.054	0.000	0.117

* Due to the shortage of participants in the studio house type, the unbalanced sample size distribution urges a cautious interpretation of the findings in this table. ** Medium to large effect size that needs to be interpreted cautiously given the sample size limitation.

**Table 3:** Regression analysis between change/novelty and public health proxy measures of AHN.

	Model Summary				ANOVA Sig.
	R	R Square	Adjusted R Square	Std. Error	
Tendency to change space used for each activity					
Depression	0.248	0.061 *	0.055	0.6899	0.003
Cognitive impairment	0.130	0.017	0.010	2.982	0.122
Anxiety	0.195	0.038 *	0.031	0.7308	0.020
Tendency to change routine					
Depression	0.152	0.023	0.016	0.7038	0.070
Cognitive impairment	0.064	0.004	−0.003	3.002	0.466
Anxiety	0.205	0.042 *	0.035	0.7292	0.014
Tendency to change the interior setting					
Depression	0.038	0.001	−0.006	0.7116	0.656
Cognitive impairment	0.084	0.007	0.000	2.997	0.321
Anxiety	0.083	0.007	0.000	0.7424	0.324

* Small effect size that needs to be interpreted cautiously given the sample size limitation.

**Table 4:** Regression analysis between residential physical activity and public health proxy measures of AHN.

	Model Summary				ANOVA Sig.
	R	R Square	Adjusted R Square	Std. Error	
Tendency to be physically active at home					
Depression	0.270	0.073 *	0.066	0.6857	0.001
Cognitive impairment	0.172	0.030 *	0.023	2.962	0.040
Anxiety	0.092	0.008	0.001	0.7419	0.278

* Small effect size that needs to be interpreted cautiously given the sample size limitation.

**Table 5:** ANOVA tests between physical activity and groups of house types.

Physical Activity at Home	ANOVA					ANOVA Effect Sizes		
	Sum of Squares (Between Groups)	df	Mean Square	F	Sig.	Point Estimate (Eta Estimate) *	Confidence Interval	
							Lower	Upper
Studio, apartment/flat, terraced house, semi-detached house, and detached house	6.659	4	1.665	2.380	0.055	0.065	0.000	0.134
Apartment/flat, terraced house, semi-detached house, and detached house	6.055	3	2.018	2.986	0.034	0.067 *	0.000	0.148
Terraced house, semi-detached house, and detached house	3.297	2	1.649	2.413	0.095	0.050	0.000	0.145

* Medium to large effect size that needs to be interpreted cautiously given the sample size limitation.

**Table 6:** Correlation analysis between physical activity, house type, and lifestyle variables.

	House Type Complexity	Getting Out of the House	Changing Space Used for Each Activity	Changing Routine	Physical Activity Throughout the House
House type complexity	1				
Getting out of the house	0.050	1			
Changing space used for each activity	0.107	0.011	1		
Changing routine	0.026	0.257 **	0.066	1	
Physical activity throughout the house	0.206 *	0.368 **	−0.051	0.238 **	1

* Correlation is significant at the 0.05 level (2-tailed). ** Correlation is significant at the 0.01 level (2-tailed).

**Table 7:** Multiple regression analysis between depression, as the dependent variable, and both house type variability and physical activity as independent variables.

	Unstandardised B	Coefficients Std. Error	Standard Coefficients Beta	t	Sig.
Four house types (apartment, terraced, semi-detached, detached) and physical activity					
Constant	2.591	0.194		13.365	<0.001
House type	−0.117	0.052	−0.189	−2.221	0.028
Physical activity	−0.232	0.072	−0.276	−3.239	0.002
Three house types (terraced, semi-detached, detached) and physical activity					
Constant	2.188	0.228		9.584	<0.001
House type	0.025	0.077	0.033	0.328	0.744
Physical activity	−0.255	0.075	−0.336	−3.386	0.001

**Table 8:** Comparison of multi-regression analysis models fit.

Model Fit	Model Summary				ANOVA	
	R	R Square	Adjusted R Square	Std. Error	F	Sig.
* 1 = Dependency of depression on the difference between four house types (apartment, terraced, semi-detached, and detached) and physical activity.	0.366	0.134	0.120	0.665	9.660	<0.001
2 = Dependency of depression on the difference between three house types (terraced, semi-detached, and detached) and physical activity.	0.333	0.111	0.092	0.607	5.743	0.004
3 = Dependency of anxiety on the difference between four house types (apartment, terraced, semi-detached, and detached) and physical activity.	0.205	0.042	0.027	0.753	2.738	0.069
4 = Dependency of cognition on the difference between four house types (apartment, terraced, semi-detached, and detached) and physical activity.	0.261	0.068	0.053	2.89	4.569	0.012
* 5 = Dependency of depression on the difference between four house types (apartment, terraced, semi-detached, and detached), physical activity, and the changingspace used for each activity.	0.406	0.165	0.145	0.655	8.178	<0.001

* Both models are significant and have large effect sizes supported with the sample size.

## Data Availability

The original contributions presented in this study are included in the article. Further inquiries can be directed to the corresponding author.
